# [Retracted] MicroRNA-365 inhibits proliferation, migration and invasion of glioma by targeting PIK3R3

**DOI:** 10.3892/or.2026.9173

**Published:** 2026-07-27

**Authors:** Yonggang Zhu, Hongguang Zhao, Min Rao, Songbai Xu

Oncol Rep 37: 2185–2192, 2017; DOI: 10.3892/or.2017.5458

Following the publication of the above article, a concerned reader drew to the Editor's attention that western blot data featured in Fig. 5D were apparently the same as those featured in a different research article by the same group in the journal American Journal of Translational Research. In addition, various of the western blot data featured in Figs. 5, 6 and 7, and the cell migration and invasion assay data in Fig. 2C and D, were strikingly similar to data that were featured in other papers written by different authors at different research institutes, some of which have since been retracted, including a pair published in 2014/2015 in the journal PLoS One.

Given the fact that the contentious data mentioned above had been included in articles published by other research groups in different journals before this paper was received at Oncology Reports, the Editor has decided that this paper should be retracted from the Journal. The authors were asked for an explanation to account for these concerns, but the Editorial Office did not receive a reply. The Editor apologizes to the readership for any inconvenience caused.

